# Predictors of Mortality in COVID-19 patients: An observational study

**DOI:** 10.12669/pjms.39.1.6059

**Published:** 2023

**Authors:** M. Arsalan Qureshi, Kaleem Ullah Toori, Raja Mobeen Ahmed

**Affiliations:** 1 Dr. M. Arsalan Qureshi, MBBS, Department of Medicine, KRL Hospital, Islamabad, Pakistan; 2 Dr. Kaleem Ullah Toori, FRCP (Glasgow), Department of Medicine, KRL Hospital, Islamabad, Pakistan; 3 Dr. Raja Mobeen Ahmed, MBBS, Department of Medicine, KRL Hospital, Islamabad, Pakistan

**Keywords:** COVID-19, Mortality, Comorbids, Inflammatory Markers, Disease severity

## Abstract

**Objectives:**

To identify the factors that affect outcome in COVID-19 patients in the Pakistani population.

**Methods:**

A total of 225 patients of COVID-19 RT-PCR proven were included during November, 2020 to June, 2021 in this cross-sectional study. They were stratified into different disease severity categories as per WHO guidelines. The characteristics of survivors and non survivors were recorded and then compared to draw conclusions.

**Results:**

Mean age was 59 years. Majority of the patients were male (68%) and the overall mortality rate was 30.1%. The non survivors were more likely to be female, had a greater number of comorbidities, had a higher respiratory rate and lower oxygen saturations at presentation and had a greater frequency of invasive mechanical ventilation. Non survivors had higher values of TLC, CRP, D-dimers and lower values of Hemoglobin and Platelets. The non survivors had higher incidence of ARDS, Septic shock and Multiorgan involvement. A higher CURB-65 score was observed in non survivors as compared to those who survived. Multivariate analysis showed that female gender, presence of and higher number of comorbid conditions and a higher CURB-65 score was linked with mortality.

**Conclusion:**

Results are compatible with international studies; increasing age, number of comorbid conditions and high inflammatory markers are associated with increased mortality. Our study had an exception that female gender had higher mortality as compared to men.

## INTRODUCTION

SARS-COV -2 originated from Wuhan China in December 2019 which was identified as a cause of new type of pneumonia. The virus has since been spread throughout the world turning into a pandemic. The WHO declared the pandemic of COVID-19 on March 11^th^, 2020.^1^ To date the virus has affected more than 300 million people and is responsible for more than 5 million deaths worldwide.^2^ Pakistan witnessed its 1^st^ case in Feb 2020.^3^ As of July 6^th^, 2021 a total of 964,490 cases with more than 22,000 deaths corresponding to about 2% mortality rate has been reported.^4^

With the virus mutating, the new Delta and Lambda variants^5^ pose a new threat to the healthcare system. The clinical presentation of the virus is not limited to one organ system and manifests with both pulmonary and extra-pulmonary features. There is a wide spectrum of symptoms ranging from simple fever and myalgia needing supportive care only to life threatening multi organ failure requiring non-invasive or invasive ventilation.^6^

Studies have recognized different parameters associated with mortality. These include increasing age, gender, presence of premorbid conditions and certain lab parameters like lymphopenia, c-reactive proteins, D-Dimers.^7^,^8^

There is paucity of data in local population regarding the determinants of mortality due to COVID-19 infection, therefore we aimed to assess potential risk factors associated with mortality in Pakistani population suffering with severe COVID-19 infection. The above would in turn help in better risk stratification and optimal allocation of resources in managing these patients in a resource constraint country.

## METHODS

It was a prospective study conducted at KRL Hospital, Islamabad which is a 350 bedded tertiary care hospital with a dedicated COVID ward and a COVID ICU, Patients were included from mid of November, 2020 to June 2021. Non probability consecutive sampling technique was used. Informed consent was taken from the patients and study was approved from the hospitals ethical committee. All patients admitted with COVID-19 PCR positive were included. Disease severity categorization was done according to WHO Living Guidance.^6^

- Non-Severe disease - Being PCR +ve, typical signs and symptoms of COVID-19 without presence of signs of Severe and Critical disease.
- Severe – in addition to above, Oxygen saturation < 90% on room air, Respiratory rate > 30 breaths/min or Signs of severe respiratory distress (accessory muscle use, inability to complete full sentences).
- Critical – presence of acute respiratory distress syndrome (ARDS), sepsis or septic shock in patients with severe COVID-19 infection.

Patients were included into the study on admission and followed up to discharge or death. Patient’s initial presenting symptoms, signs, lab parameters, co-morbids, scores for pneumonia severity such as CURB-65 and Sequential Organ Failure Assessment (SOFA) scores were recorded and analyzed using SPSS version 23. Chi square test was used to compare categorical data and Student’s t-test was used to compare continuous data. Univariate binary logistic regression was used for modelling primary outcome with initial patient variables and all variables with p<0.25 were considered for multivariate binary logistic regression.

## RESULTS

A total of 225 patients were included in the study. The mean age was 59.3 years with 68% (153/225) male and 39.1% (88/225) of patients died while 60.9% (137/225) survived and discharged home. The baselines characteristics of survivors and non-survivors are compared in Table-I. Non-survivors were older (mean age 64.4 years) as compared to survivors (mean age 56.0 years) and this difference was statistically significant (p<0.001). Females were more likely to die as compared to males (52.8% vs 32.7%, p<0.05).

**Table-I:** Baseline Clinical and Laboratory Parameters.

	Survivor’s n (%)	Non-survivors n (%)	Overall n (%)	p-value
Age *(years)	56.0±11.1	64.4±10.5	59.3±11.6	<0.001
***Gender***				0.004
Male	103 (75.2)	50 (56.8)	153 (68.00)	
Female	34 (24.8)	38 (43.2)	72 (32.0)	
***Symptoms***				
Fever	116 (84.7)	78 (88.6)	194 (86.2)	0.400
Cough	91 (61.4)	60 (68.2)	151 (67.1)	0.784
Shortness of breath	104 (75.9)	75 (85.2)	179 (79.6)	0.091
Myalgia	32 (23.4)	15 (17.0)	47 (20.9)	0.256
Headache	2 (1.5)	0 (0.00	2 (0.9)	0.255
Sore Throat	11 (8.0)	2 (2.3)	13 (5.8)	0.071
Rhinorrhea	1 (0.7)	2 (2.3)	3 (1.3)	0.325
Hemoptysis	2 (1.5)	0 (0.0)	2 (0.9)	0.255
***Symptom Duration*** **	6 (4-9)	6 (4-8)	6 (4-8)	0.918
***Co-morbids***				
Hypertension	64 (46.7)	53 (60.20)	117 (52.0)	0.048**
Diabetes	66 (48.2)	48 (54.5)	114 (50.7)	0.351
Chronic Kidney Disease	7 (5.1)	6 (8.0)	14 (6.2)	0.389
Chronic Liver Disease	4 (2.9)	7 (8.0)	11 (4.9)	0.087
Cardiovascular Diseases	18 (13.1)	15 (17.0)	33 (14.7)	0.419
Chronic Respiratory Diseases	13 (9.5)	20 (22.7)	33 (14.7)	0.006
Chronic Neurological Disease	3 (2.2)	5 (5.7)	8 (3.6)	0.167
Cancer	1 (0.7)	4 (4.5)	5 (2.2)	0.058
***Number of Co-morbids***				0.075
0	38 (27.7)	13 (14.8)	51 (22.7)	
1	37 (27.0)	27 (30.7)	64 (28.4)	
2 or more	62 (45.3)	48 (54.5)	110 (48.9)	
***Presenting Vitals***				
Heart Rate** (/min)	92 (87-100)	98 (87-110)	95 (88-103)	0.086
Blood pressure				0.265
Normal	91 (61.4)	52(59.1)	143 (63.6)	
Abnormal	46 (33.6)	36(40.1)	82 (36.4)	
Respiratory Rate** (/min)	24 (21-26)	28 (22-30)	24 (22-28)	<0.001
Temperature** (°F)	98.6 (98.6-100.0)	98.6 (98.6-100.0)	98.6 (98.6-100.0)	0.337
Oxygen Saturation** (%)	90 (86-92)	84 (75-89)	88 (82-91)	<0.001
***Symptom Severity***				<0.001
Moderate	22 (16.1)	7 (8)	29 (12.9)	
Severe	87 (63.5)	31 (35.2)	118 (52.4)	
Critically Ill	28 (20.4)	50 (56.8)	78 (34.7)	
***Initial Lab values***				
Hemoglobin **(g/dL)	13.4 (12.1-14.3)	13.1 (11.8-14.8)	13.2 (12.0-14.5)	0.849
Platelets **(x10^9/L)	212 (150-270)	211 (161-263)	211 (150-264)	0.804
Total leukocyte count **(x10^9/L)	7.9 (6.3-10.8)	10.5 (7.1-12.9)	8.8 (6.6-11.9)	0.006
Lymphocytes (%)	14 (9-21)	10 (7-17)	12 (8-20)	0.011
Neutrophils (%)	79 (70-85)	82 (74-88)	80 (72-86)	0.004
NLR	5.8 (3.2-10.3)	8.1 (4.1-12.4)	6.9 (3.7-11.2)	0.803
Lymphopenia	119 (86.9)	85 (96.7)	204 (90.7)	0.038
C-Reactive protein **(mg/L)	98.6 (43.5-150.7)	123.0 (65.2-187.9)	106.0 (49.5-160.5)	0.014
D-dimers **(ng/ml)	544 (291-1251)	741 (447-2443)	629 (327-1582)	0.005
Ferritin **(ng/ml)	695 (342-1052)	879 (554-1456)	732 (421-1159)	0.056
Lactate dehydrogenase **(U/L)	353 (283-550)	509 (408-676)	422 (303-609)	<0.001
Pro-calcitonin **(ng/ml)	0.13 (0.08-0.62)	0.20 (0.10-0.94)	0.18 (0.09-0.77)	0.215
***CURB-65*** **	0 (0-1)	1 (1-2)	1 (0-1)	<0.001
***SOFA score*** **	2 (2-3)	3 (2-4)	2 (2-3)	<0.001

* Mean ±SD

** Median (Interquartile Range), NLR: Neutrophil to Lymphocyte Ratio, CURB-65: Confusion, Urea nitrogen, Respiratory rate, Blood pressure, 65 years of age and older, SOFA: Sequential Organ Failure Assessment.

The most common presenting symptoms (Table-I) were fever (86.2%), shortness of breath (79.6%) and dry cough (67.1%) with no significant difference between survivors and non-survivors. Majority of the patients (52.4%) had severe disease and greater disease severity was associated with mortality (p<0.001). Majority of patients had two or more co-morbid conditions and the most common co-morbid conditions were hypertension and diabetes. Among comorbids, hypertension and chronic respiratory diseases were significantly associated with mortality (p=0.048 & p=0.006 respectively).

On presentation, both groups had similar pulse rates, temperature and blood pressures, but non-survivors had a greater respiratory rate (27/min vs 24/min, p<0.001) and lower oxygen saturation while breathing room air (80.1% vs 88.1%, p<0.001) as compared to survivors.

The laboratory tests done at admission (Table-I) revealed similar hemoglobin, platelets, neutrophil-to-lymphocyte ratio (NLR), ferritin and procalcitonin levels in both groups. Non-survivors had a higher C-reactive protein (139.9 vs 109.6 mg/L, p<0.05), higher total leucocyte count (10640 vs 9020 x10^9/L, p<0.05) with raised neutrophils (8075 vs 7668 x10^9/L, p<0.05) and lower lymphocytes (1239 vs 1527 x10^9/L, p<0.05), higher Lactate Dehydrogenase (564 vs 424 U/L, p<0.001), higher D-dimers (2079 vs 1197 ng/ml, p<0.05), and higher number of patients with lymphopenia n= 204 (p<0.05), deranged renal function tests (51 vs 25 p<0.001), deranged liver function tests (25 vs 7 p<0.001) and deranged coagulation profiles (p<0.05) as compared to survivors. Also, higher CURB-65 and SOFA scores on admission were significantly associated with mortality (p<0.001 & p<0.05 respectively).

In labs done during hospital course (Table-II), a higher neutrophil count (p<0.001) was associated with mortality. Oxygen dependency (Table-II), use of non-invasive ventilation and mechanical ventilation was associated with increase in mortality (p<0.05). Only 11.1% of the mechanically ventilated patients survived and duration of ventilator support did not have association with survival (8.7 days in survivors vs 7.8 days in non-survivors, p>0.05). Development of Acute Respiratory Distress Syndrome (p<0.001), Septic Shock (p<0.001) and multi-organ failure (p<0.001) was associated with mortality (Table-II).

**Table-II:** Labs during Hospital Course, Use of Oxygen and Complications.

Labs	Survivors n(%)	Non-survivors n(%)	Overall n(%)	p-value
Hemoglobin **(g/dL)	12.5 (11.2-14.0)	12.2 (10.8-13.7)	12.3 (11.0-13.9)	0.560
Platelets **(x10^9/L)	273 (174-386)	169 (116-242)	215 (148-341)	<0.001
Total leukocyte count **(x10^9/L)	9.8 (7.1-11.4)	16.7 (11.5-21.4)	11.2 (8.8-16.3)	<0.001
Lymphocytes (%)	15 (8-21)	5 (4-8)	9 (5-18)	<0.001
Neutrophils (%)	75 (68-80)	90 (87-92)	80 (74-89)	<0.001
NLR	4.8 (3.1-9.1)	15 (8.2-22.3)	7.3 (3.8-15.4)	<0.001
C-Reactive protein **(mg/L)	20.8 (7.3-51.5)	132.0 (40.1-225.5)	40.7 (11.6-104.5)	<0.001
D-dimers **(ng/ml)	500 (329-988)	4753 (1745-8499)	1139 (440-4973)	<0.001
Renal Function Tests				<0.001
Normal	112 (81.8)	38 (43.2)	149 (66.2)	
Abnormal	25 (18.3)	51 (58.0)	76 (33.8)	
Liver Function Tests				<0.001
Normal	130 (94.9)	63 (71.6)	193 (85.8)	
Abnormal	7 (5.1)	25 (28.4)	32 (14.2)	
Oxygen Therapy				
Yes	122 (89.1)	87 (98.9)	209 (92.9)	0.001
No	15 (10.9)	1 (1.1)	16 (7.1)	
Non-Invasive Ventilation	17 (12.4)	54 (62.1)	71 (31.6)	<0.001
Mechanical Ventilation	6 (4.4)	48 (54.5)	54 (24)	<0.001
Ventilation Duration **	8 (6-12)	6 (2-11)	6 (3-11)	0.772
Complications				
ARDS	26 (19.0)	75 (86.2)	101 (45.3)	<0.001
Septic Shock	3 (2.2)	36 (41.4)	39 (17.4)	<0.001
Multi-Organ Failure	2 (1.5)	41 (47.1)	43 (19.2)	<0.001

*Mean ±SD,

** Median (Interquartile Range), NLR: Neutrophil to Lymphocyte Ratio, ARDS: Acute respiratory distress syndrome.

Univariate binary logistic regression analysis of all variables was done and variables with a p value <0.25 were selected for multivariate binary logistic regression (Table-III). The final model showed that female gender, chronic liver disease, underlying cancer, chronic respiratory disease, higher total number of comorbids and higher CURB-65 scores predicted increased odds of mortality (Table-IV).

**Table-III:** Univariate Binary Logistic Regression.

Variables	Crude OR	95% CI	p-value
Age	1.077	1.046-1.109	<0.001
Gender (female)	2.302	1.298-4.083	0.004
Fever	1.412	0.631-3.161	0.401
Cough	1.083	0.611-1.919	0.784
Shortness of Breath	1.831	0.903-3.713	0.094
Myalgia	0.674	0.341-1.334	0.257
Headache	0.000	0.000	0.999
Sore Throat	0.266	0.058-1.232	0.090
Hemoptysis	0.000	0.000	0.999
Rhinorrhea	3.163	0.282-35.412	0.350
Symptoms Duration	0.996	0.925-1.072	0.917
Hypertension	1.727	1.003-2.973	0.049
Diabetes	1.291	0.755-2.209	0.351
Chronic Kidney Disease	1.605	0.543-4.744	0.392
Chronic Liver Disease	2.873	0.816-10.122	0.100
Cardiovascular Disease	1.358	0.645-2.860	0.420
Cancer	6.476	0.712-58.924	0.097
Chronic Respiratory Disease	2.805	1.314-5.989	0.008
Chronic Neurological Condition	2.691	0.627-11.555	0.183
Number of comorbids	1.424	1.009-2.010	0.044
Symptoms Severity	3.192	1.982-5.141	<0.001
Pulse	1.016	0.998-1.035	0.088
Temperature	1.108	0.899-1.366	0.336
Blood pressure	0.730	0.420-1.270	0.265
Oxygen Saturation	0.924	0.894-0.955	<0.001
Respiratory Rate	1.128	1.060-1.199	<0.001
Hemoglobin	0.988	0.869-1.122	0.848
C-reactive protein	1.004	1.001-1.007	0.017
Total leukocyte count	1.091	1.023-1.163	0.008
Lymphocytes	0.956	0.922-0.990	0.012
Neutrophils	1.043	1.013-1.073	0.005
Lymphopenia	3.571	1.003-12.723	0.050
Platelets	1.000	0.997-1.002	0.767
NLR	0.999	0.998-1.010	0.804
Lactate dehydrogenase	1.003	1.001-1.004	<0.001
D-dimers	1.000	1.000-1.000	0.008
Ferritin	1.000	1.000-1.000	0.080
Procalcitonin	1.034	0.966-1.108	0.337
CURB-65	4.375	2.876-6.654	<0.001
SOFA	1.459	1.181-1.802	<0.001

NLR: Neutrophil to Lymphocyte Ratio, CURB-65: Confusion, Urea nitrogen, Respiratory rate, Blood pressure, 65 years of age and older. SOFA: Sequential Organ Failure Assessment.

**Table-IV:** Multivariate binary logistic regression.

Variables	Odds Ratio	95% CI	p-value
Age	0.987	0.941-1.035	0.585
Gender	0.221	0.082-0.595	0.003
Shortness of Breath	1.810	0.554-5.915	0.326
Sore Throat	2.050	0.244-17.240	0.509
Hypertension	0.269	0.068-1.061	0.061
Chronic Liver Disease	0.102	0.014-0.715	0.022
Underlying Cancer	0.013	0.001-0.953	0.048
Chronic Respiratory Disease	0.105	0.027-0.402	0.001
Chronic Neurological Condition	0.739	0.032-16.974	0.850
Number of comorbids	8.313	2.065-33.469	0.003
Symptoms Severity	0.463	0.133-1.613	0.227
Pulse	1.011	0.980-1.043	0.481
Oxygen Saturation	0.964	0.904-1.027	0.257
Respiratory Rate	0.934	0.835-1.046	0.238
C-reactive protein	1.002	0.996-1.007	0.538
Total Leukocyte count	1.037	0.905-1.188	0.603
Lymphocytes	0.990	0.867-1.131	0.883
Neutrophils	1.027	0.933-1.129	0.590
Lymphopenia	0.635	0.043-9.397	0.741
Lactate dehydrogenase	1.001	0.998-1.003	0.678
D-dimers	1.000	1.000-1.000	0.650
Ferritin	1.000	1.000-1.000	0.883
CURB-65	0.026	0.002-0.404	0.009
SOFA	0.873	0.003-263.647	0.963

CURB-65: Confusion, Urea nitrogen, Respiratory rate, Blood pressure, 65 years of age and older. SOFA: Sequential Organ Failure Assessment.

## DISCUSSION

In this study we report the risk factors which can predict mortality in COVID-19 patients with the view to do risk stratification at the time of admission and to optimally use the resources in managing seriously ill patients in resource constraint environment. We found that female gender, increasing age, high respiration rate, increased inflammatory markers, presence of comorbid conditions, and high CURB 65 score are associated with poor outcomes in our study population.

We observed significantly increased mortality among female COVID-19 patients as compared to males which is contrary to the available evidence.^9^ Part of this can be attributed to local social norms; where women prefer to be treated on outpatient basis and to only get admitted in hospital when can’t cope at home. Consequently, the admitted female population has relatively more severe form of disease at presentation as compared to males and hence increased mortality. We suggest studies with a larger sample size to further probe this finding.

COVID-19 has propensity to affect male gender more than females.^9^ The virus’s bias against the male gender is due to a widely accepted hypothesis that there is a difference in ACE-2 receptors between men and women; men tend to have higher ACE-2 receptors as compared to women, hence more potential entry sites for the coronavirus.^10^ Our study population comprised mostly of males (68%) which is in keeping with the available evidence as above.

Advancing age^11^ is usually associated with increasing number of co morbid conditions i.e., hypertension, diabetes, ischemic heart disease etc. Research from the US involving more than half a million hospitalized patients confirmed a positive correlation between the increasing number underlying medical conditions and risk of developing severe COVID -19 and hence a higher chance death.^12^ Furthemore it was reported that higher age groups had a higher disease severity. Our analysis also revealed that non-survivors consisted of higher age group as compared to survivors.

As in the WHO’s guidelines^3^ and a review comparing COVID-19 symptoms around the globe,^13^ we also found fever, cough and shortness of breath to be the most common symptoms in our study population. It is important to assess and triage patients at the time of admission so as to identify the high-risk group and to promptly start their management. Vital signs make an essential component of this assessment. We found increasing respiratory rate and higher degree of hypoxia at presentation as markers of poor prognosis in our study and these findings are well supported by the literature.^7^,^14^,^15^

The CURB-65^16^ and SOFA 17 scores are validated tools to assess pneumonia and ICU mortality respectively. We employed these scores in our study population and univariate analysis showed significantly higher CURB-65 and SOFA scores in non-surviving patients. A regional study from Karachi, Pakistan concurred similar findings,^15^ also multiple international studies have shown high CURB 65 and SOFA scores predicting mortality with good efficacy in COVID-19 infection.^18^-^21^

Presence of comorbidities is an established risk factor for poor outcome in various medical conditions^22^-^24^ and it holds true for COVID-19 as well. Recently, data from two large cohorts published by the American Center for Disease Control and Prevention concluded that the presence of comorbid conditions pose a significant in-hospital mortality risk in patients affected by the Corona virus.^25^ We found the presence of hypertension, chronic respiratory disease, chronic liver disease and cancer as risk factors for mortality in our study. We also studied the effect of increasing number of comorbidities on mortality and found it to be positively associated with it. A review involving over 20,000 patients^26^ and another study by Gujski M et al.^27^ support our above finding of having higher odds of mortality with increasing number of comorbid conditions.

COVID-19 causes a systemic inflammatory response leading to release of various inflammatory biomarkers in the body. Escalating levels of inflammatory biomarkers lead to increasing severity of COVID-19 illness.^28^ Poor clinical outcomes including mortality have been reported with high levels of inflammatory biomarkers in various studies. Ali N et al. studied various inflammatory biomarkers in predicting complications and in-hospital mortality in patients with COVID-19 and reported good predictable value of serum LDH and D-Dimers levels in this regard.^29^ Another study from the Indus Hospital, Karachi showed poor clinical outcome with raised CRP and D-Dimers levels.^15^ Our results concur with above and show association of high CRP, D-Dimer, LDH, and Ferritin levels with mortality in COVID-19 infection.

Recently, interest has been shown in checking Neutrophil to Lymphocyte ratio (NLR) in various medical conditions with the notion to identify its role as prognostic marker.^30^-^32^ Being cheap and easily performable tool, it has been widely studied during COVID-19 pandemic as well so as to employ it in triaging patients at the time of admission.^33^,^34^ Current study shows that high NLR is associated with increased risk of mortality which is further endorsed by our group previously published study highlighting the importance of NLR in COVID-19 patients in predicting disease severity and mortality.^34^ In addition, Pervaiz et al. has further shown its role in predicting the need for mechanical ventilation in severe COVID-19 patients.^3^

Another important laboratory finding in COVID-19 patients is thrombocytopenia which is primarily caused by viral infiltration of the bone marrow with consequent suppressing the ability of megakaryocytes to release platelets. In critical disease it is further enhanced by development of disseminated intravascular coagulopathy (DIC). We have shown worsening thrombocytopenia association with higher mortality risk which is in line with published data from Wuhan, China.^36^

Development of ARDS is a recognized complication of COVID-19 and may require high flow oxygen, non-invasive or invasive ventilation. We looked at mortality figures in above category of patients and found that only 11% of patients needing invasive mechanical ventilation survived, leading to the conclusion that high mortality is associated with patients who require mechanical ventilation. Varying survival/mortality percentages have been reported in literature; an American study reported 3% recovery from invasive mechanical ventilation^37^, a study conducted at Tongji Hospital, China reported 3% recovery from invasive ventilation,^38^ and an Italian study reported a high mortality reaching 78% with the use of invasive mechanical ventilation.^39^

### Limitations:

Our prediction towards the female gender should be investigated further with a large sample sized study. We conducted a single center study perhaps a multicentered study with large sample size would further authenticate the findings and hence their application to general population. The major strength of the study is the use of simple clinical and laboratory parameters as tool to predict COVID-19 mortality and its applicability in triaging patients at time of admission.

## CONCLUSION

Patients with COVID-19 illness may progress to severe disease associated with significant morbidity and mortality. Female gender, increasing age, high respiration rate, increased inflammatory markers, presence of comorbid conditions, and high CURB 65 score at presentation are risk factors associated with poor outcomes including mortality. Identifying above risk factors and triaging patients at presentation may help in better management and improving the final outcome.

### Authors Contribution:

**MAQ:** Conceived, designed, contributed to data collection and manuscript writing.

**KT:** Did statistical analysis along with final review of manuscript.

**RMA:** Contributed to data collection and manuscript writing.

## References

[1] Cucinotta D, Vanelli M (2020). WHO declares COVID-19 a pandemic. Acta Biomed.

[2] World Health Organization (2021). WHO covid19.who.int/.

[3] World Health Organization (2020). WHO extends support to Pakistan as it confirms its first two cases of Covid-19 [Internet] emro.who.int.com.

[4] The Government of Pakistan Covid-19 Dashboard [Internet] covid.gov.pk.

[5] The Daily Mail World's MOST transmissible Covid variant'has now been spotted in 31 countries:Scientists baffled why 'more lethal, more infectious'Lambda strain isn't taking over from Delta. [Internet] Dailymail.co.uk.

[6] WHO COVID-19 (2021). Clinical management living guideline.

[7] Lu Y, Huang Z, Wang M, Tang K, Wang S, Gao P (2021). Clinical characteristics and predictors of mortality in young adults with severe COVID-19:A retrospective observational study. Ann Clin Microbiol Antimicrob.

[8] Popadic V, Klasnja S, Milic N, Rajovic N, Aleksic A, Milenkovic M (2021). Predictors of Mortality in Critically Ill COVID-19 Patients Demanding High Oxygen Flow:A Thin Line between Inflammation, Cytokine Storm, and Coagulopathy. Oxid Med Cell Longev.

[9] Chen T, Wu DI, Chen H, Yan W, Yang D, Chen G (2020). Clinical characteristics of 113 deceased patients with coronavirus disease 2019:Retrospective Study. BMJ.

[10] Li W, Zhang C, Sui J, Kuhn JH, Moore MJ, Luo S (2005). Receptor and viral determinants of SARS?coronavirus adaptation to human ACE2. EMBO J.

[11] Muhammad R, Ogunti R, Ahmed B, Munawar M, Donaldson S, Sumon M (2022). Clinical characteristics and predictors of mortality in minority patients hospitalized with COVID-19 infection. J Racial Ethn Health Disparities.

[12] Kompaniyets L, Pennington AF, Goodman AB, Rosenblum HG, Belay B, Ko JY (2021). Peer Reviewed:Underlying Medical Conditions and Severe Illness Among 540,667 Adults Hospitalized With COVID-19, March 2020-March 2021. Prev Chronic Dis.

[13] Noor AU, Maqbool F, Bhatti ZA, Khan AU (2020). Epidemiology of CoViD-19 Pandemic:Recovery and mortality ratio around the globe. Pak J Med Sci.

[14] Chen G, Wu DI, Guo W, Cao Y, Huang D, Wang H (2020). Clinical and immunological features of severe and moderate coronavirus disease 2019. J Clin Invest.

[15] Sarfaraz S, Shaikh Q, Saleem SG, Rahim A, Herekar FF, Junejo S (2021). Determinants of in-hospital mortality in COVID-19;A prospective cohort study from Pakistan. Plos One.

[16] Lim WS, Van der Eerden MM, Laing R, Boersma WG, Karalus N, Town GI, Lewis SA, Macfarlane J (2003). Defining community acquired pneumonia severity on presentation to hospital:an international derivation and validation study. Thorax.

[17] Vincent JL, Moreno R, Takala J, Willatts S, De Mendonça A, Bruining H (1996). The SOFA (Sepsis-related Organ Failure Assessment) score to describe organ dysfunction/failure. Intensive Care Med.

[18] Yang Z, Hu Q, Huang F, Xiong S, Sun Y (2021). The prognostic value of the SOFA score in patients with COVID-19:a retrospective, observational study. Medicine (Baltimore).

[19] Liu S, Yao N, Qiu Y, He C (2020). Predictive performance of SOFA and qSOFA for in-hospital mortality in severe novel coronavirus disease. Am J Emerg Med.

[20] Nguyen Y, Corre F, Honsel V, Curac S, Zarrouk V, Fantin B (2020). Applicability of the CURB-65 pneumonia severity score for outpatient treatment of COVID-19. J Infect.

[21] Satici C, Demirkol MA, Altunok ES, Gursoy B, Alkan M, Kamat S (2020). Performance of pneumonia severity index and CURB-65 in predicting 30-day mortality in patients with COVID-19. Int J Infect Dis.

[22] Alqahtani FY, Aleanizy FS, Mohamed RA, Alanazi MS, Mohamed N, Alrasheed MM (2019). Prevalence of comorbidities in cases of Middle East respiratory syndrome coronavirus:a retrospective study. Epidemiol Infect.

[23] Holguin F, Folch E, Redd SC, Mannino DM (2005). Comorbidity and mortality in COPD-related hospitalizations in the United States, 1979 2001. Chest.

[24] Sachdev M, Sun JL, Tsiatis AA, Nelson CL, Mark DB, Jollis JG (2004). The prognostic importance of comorbidity for mortality in patients with stable coronary artery disease. J Am Coll Cardiol.

[25] Centers for Disease Control and Prevention Underlying medical conditions associated with high risk for severe COVID-19:Information for healthcare providers.

[26] Bajgain KT, Badal S, Bajgain BB, Santana MJ (2021). Prevalence of comorbidities among individuals with COVID-19:A rapid review of current literature. Am J Infect Control.

[27] Gujski M, Jankowski M, Rabczenko D, Gorynski P, Juszczyk G (2021). Characteristics and Clinical Outcomes of 116,539 Patients Hospitalized with COVID-19-Poland, March-December 2020. Viruses [Internet]. MDPI AG.

[28] Inflammatory Markers Huang I, Pranata R, Lim MA, Oehadian A, Alisjahbana B (2020). C-reactive protein, procalcitonin, D-dimer, and ferritin in severe coronavirus disease-2019:a meta-analysis. Therapeutic advances in respiratory disease.

[29] Ali N, Kapadia NN, Aymen D, Baig N (2022). Utility of biomarkers in predicting complications and in-hospital mortality in patients with COVID-19. Pak J Med Sci.

[30] Guthrie GJ, Charles KA, Roxburgh CS, Horgan PG, McMillan DC, Clarke SJ (2013). The systemic inflammation-based neutrophil-lymphocyte ratio:experience in patients with cancer. Criti Rev Oncol Hematol.

[31] Azab B, Zaher M, Weiserbs KF, Torbey E, Lacossiere K, Gaddam S (2010). Usefulness of neutrophil to lymphocyte ratio in predicting short-and long-term mortality after non-ST-elevation myocardial infarction. Am J Cardiol.

[32] Ha YJ, Hur J, Go DJ, Kang EH, Park JK, Lee EY (2018). Baseline peripheral blood neutrophil-to-lymphocyte ratio could predict survival in patients with adult polymyositis and dermatomyositis:A retrospective observational study. PLoS One.

[33] Chan AS, Rout A (2020). Use of neutrophil-to-lymphocyte and platelet-to-lymphocyte ratios in COVID-19. J Clin Med Res.

[34] Toori KU, Qureshi MA, Chaudhry A, Safdar MF (2021). Neutrophil to lymphocyte ratio (NLR) in COVID-19:A cheap prognostic marker in a resource constraint setting. Pak J Med Sci.

[35] Pervaiz A, Pasha U, Bashir S, Arshad R, Waseem M, Qasim O (2020). Neutrophil to lymphocyte ratio (NLR) can be a predictor of the outcome and the need for mechanical ventilation in patients with COVID-19 in Pakistan. Pak J Pathol.

[36] Yang X, Yang Q, Wang Y, Wu Y, Xu J, Yu Y (2020). Thrombocytopenia and its association with mortality in patients with COVID?19. J Thromb Haemost.

[37] Richardson S, Hirsch JS, Narasimhan M, Crawford JM, McGinn T, Davidson KW (2020). Presenting characteristics comorbidities, and outcomes among 5700 patients hospitalized with COVID-19 in the New York City area. JAMA.

[38] Wang Y, Lu X, Li Y, Chen H, Chen T, Su N (2020). Clinical course and outcomes of 344 intensive care patients with COVID-19. Am J Respir Crit Care Med.

[39] Grasselli G, Greco M, Zanella A, Albano G, Antonelli M, Bellani G (2020). Risk factors associated with mortality among patients with COVID-19 in intensive care units in Lombardy Italy. JAMA Intern Med.

